# Supplementary material for: Novel Driver Strength Index highlights important cancer genes in TCGA PanCanAtlas patients
Source: PeerJ. 2022 Aug 11;10:e13860. doi: 10.7717/peerj.13860 (PMC9375969; doi:10.7717/peerj.13860)
Supplement: Supplemental Information 2 [file peerj-10-13860-s002.zip › COHORTS/genes plots/detailed/2021_8_16_14_9_distribution_events_detailed_males_3.pdf]

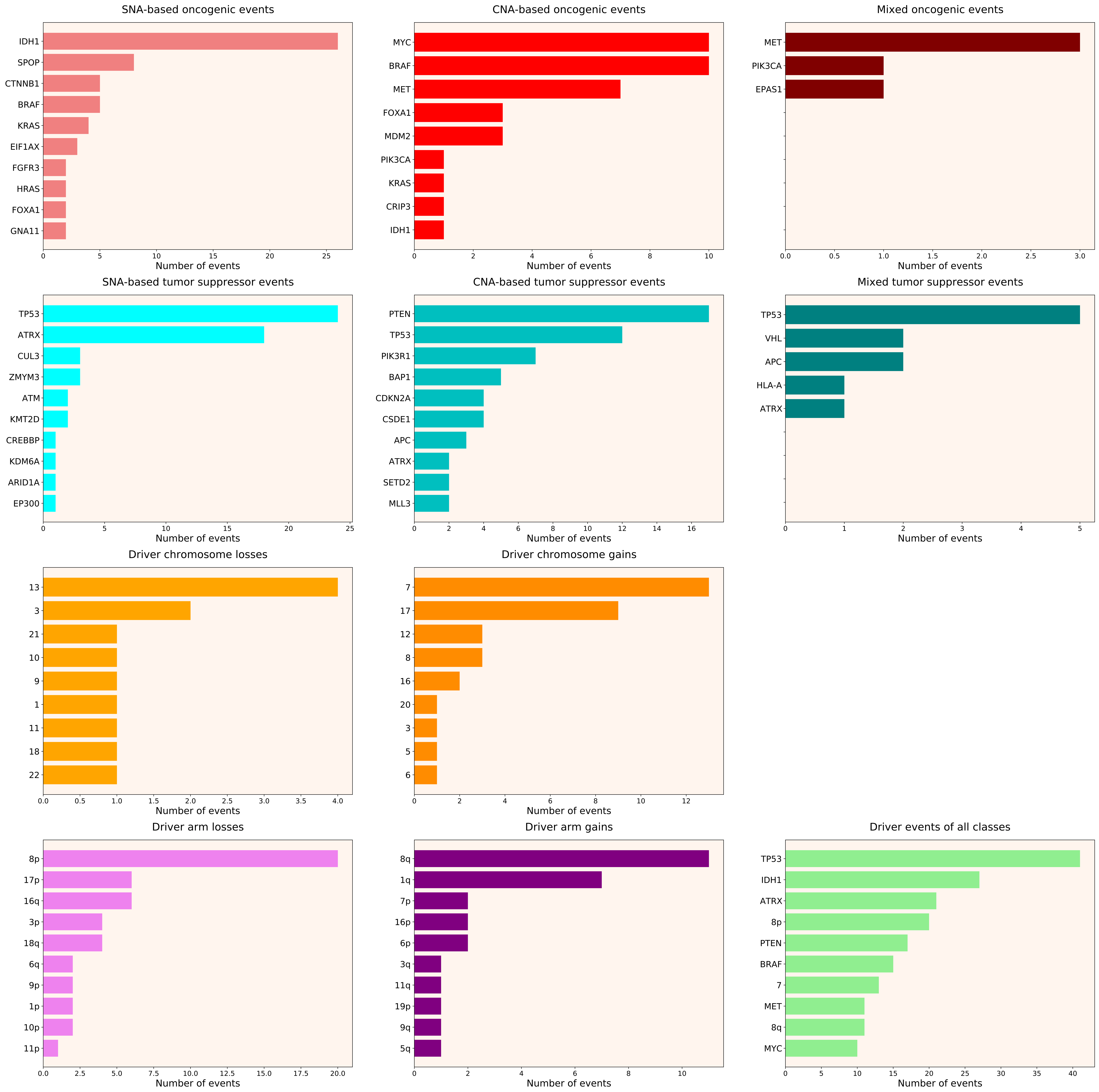
SNA-based oncogenic events
IDH1
SPOP
CTNNB1
BRAF
KRAS
EIF1AX
FGFR3
HRAS
FOXA1
GNA11
0 5 10 15 20 25
Number of events
CNA-based oncogenic events
MYC
BRAF
MET
FOXA1
MDM2
PIK3CA
KRAS
CRIP3
IDH1
0 2 4 6 8 10
Number of events
Mixed oncogenic events
MET
PIK3CA
EPAS1
0.0 0.5 1.0 1.5 2.0 2.5 3.0
Number of events
SNA-based tumor suppressor events
TP53
ATRX
CUL3
ZMYM3
ATM
KMT2D
CREBBP
KDM6A
ARID1A
EP300
0 5 10 15 20 25
Number of events
CNA-based tumor suppressor events
PTEN
TP53
PIK3R1
BAP1
CDKN2A
CSDE1
APC
ATRX
SETD2
MLL3
0 2 4 6 8 10 12 14 16
Number of events
Mixed tumor suppressor events
TP53
VHL
APC
HLA-A
ATRX
0 1 2 3 4 5
Number of events
Driver chromosome losses
13
3
21
10
9
1
11
18
22
0.0 0.5 1.0 1.5 2.0 2.5 3.0 3.5 4.0
Number of events
Driver chromosome gains
7
17
12
8
16
20
3
5
6
0 2 4 6 8 10 12
Number of events
Driver arm losses
8p
17p
16q
3p
18q
6q
9p
1p
10p
11p
0.0 2.5 5.0 7.5 10.0 12.5 15.0 17.5 20.0
Number of events
Driver arm gains
8q
1q
7p
16p
6p
3q
11q
19p
9q
5q
0 2 4 6 8 10
Number of events
Driver events of all classes
TP53
IDH1
ATRX
8p
PTEN
BRAF
7
MET
8q
MYC
0 5 10 15 20 25 30 35 40
Number of events
